# Acute Pancreatitis as the Initial Presentation of Metastatic Breast Cancer Due to Malignant Hypercalcemia: A Case Report

**DOI:** 10.7759/cureus.104627

**Published:** 2026-03-03

**Authors:** Jaysoom W Abarca Ruiz, Mauricio N Suárez Caicedo, Edgar J Redroban Tufino, Estefanía A Arteaga Morocho, Patricia H Corella Sanguil

**Affiliations:** 1 Gastroenterology, Hospital de Especialidades Eugenio Espejo, Quito, ECU; 2 Gastroenterology, Pontificia Universidad Católica del Ecuador, Quito, ECU

**Keywords:** acute pancreatitis, bone metastasis, breast neoplasms, hypercalcemia, multiple myeloma

## Abstract

Hypercalcemia-induced acute pancreatitis is a rare condition, most commonly associated with primary hyperparathyroidism or advanced malignancies, and represents a metabolic emergency.

We report the case of a 63-year-old woman who presented with severe epigastric abdominal pain, constipation, and episodes of disorientation. Laboratory tests revealed elevated pancreatic enzymes and severe hypercalcemia. Acute pancreatitis secondary to hypercalcemia was diagnosed, and normal parathyroid hormone (PTH) levels excluded primary hyperparathyroidism. The presence of anemia, thrombocytopenia, and multiple lytic bone lesions initially suggested multiple myeloma; however, this diagnosis was ruled out due to the absence of monoclonal gammopathy. Further imaging revealed an irregular right breast mass with axillary lymphadenopathy, classified as BI-RADS 5. Tumor markers were markedly elevated, and core needle biopsy confirmed human epidermal growth factor receptor 2 (HER2)-positive invasive ductal carcinoma with bone metastases. Despite intensive treatment with intravenous hydration and zoledronic acid, neurological deterioration occurred; hence, after 25 days of hospitalization, the patient was discharged for home-based palliative care at the family’s request.

This case highlights the importance of considering metastatic malignancies, particularly breast cancer, in patients with pancreatitis of unclear etiology and severe hypercalcemia.

## Introduction

Acute pancreatitis is an inflammatory condition of the pancreas, characterized by intraglandular activation of digestive enzymes, leading to tissue necrosis, systemic inflammatory response, and, in severe cases, multiorgan failure [1]. The most common causes are gallstone disease and excessive alcohol consumption, accounting for more than 70% of cases. However, less frequent etiologies exist, among which hypercalcemia represents a metabolic factor present in fewer than 8% of cases and is generally associated with primary hyperparathyroidism or advanced malignant neoplasms [1-3].

Malignancy-related hypercalcemia occurs in approximately 20%-30% of patients with metastatic cancer and is considered a metabolic emergency. Its pathophysiology includes secretion of parathyroid hormone (PTH) or parathyroid hormone-related peptide (PTHrP), osteolytic bone metastases, and increased production of 1,25-dihydroxyvitamin D [2-4]. In these settings, excessive calcium causes direct pancreatic injury through intracellular calcium dysregulation, premature trypsin activation, and microlith formation within pancreatic ducts, which in turn promotes premature activation of pancreatic enzymes and inflammatory injury [2,5,6].

Although hypercalcemia is relatively common in patients with bone metastases, its presentation as the initial manifestation of acute pancreatitis is rare and poses a diagnostic challenge. Few cases of malignancy-related, hypercalcemia-induced pancreatitis have been reported, most of which describe patients with a previously known cancer diagnosis [7]. Among malignant causes, breast cancer is an uncommon initial cause of hypercalcemia-induced pancreatitis, making this presentation particularly unusual; meanwhile, multiple myeloma is particularly frequent, and differentiation between these entities is essential, as it directly impacts management, prognosis, and therapeutic options [2,3].

In this context, we present a case of acute pancreatitis secondary to malignant hypercalcemia, highlighting the importance of multidisciplinary evaluation and clinical reasoning in uncommon etiologies of acute pancreatitis.

## Case presentation

A 63-year-old mestizo woman, homemaker, born and residing in Quito, living with her family (providing adequate social support), with basic education and of Catholic faith, denied alcohol, tobacco, or illicit drug use. She reported a balanced diet with three main meals per day and restorative sleep. Her medical history included hepatic steatosis and laparoscopic cholecystectomy. There was no family history of malignancy or relevant genetic or metabolic diseases.

She presented with a five-day history of severe epigastric abdominal pain, radiating to the lumbar region and accompanied by asthenia, constipation, and intermittent episodes of disorientation. On physical examination, she was conscious and oriented, with pale mucous membranes. The abdomen was soft and depressible, with tenderness in the epigastrium and flanks, without peritoneal signs.

Initial laboratory findings demonstrated elevated pancreatic enzymes (lipase more than 10 times the upper reference limit), severe hypercalcemia (serum calcium ≥14 mg/dL), anemia, thrombocytopenia, mild renal dysfunction, and increased inflammatory and tissue injury markers. Hepatobiliary enzymes showed mild aspartate aminotransferase (AST) elevation, with mildly elevated gamma-glutamyl transferase (GGT) and alkaline phosphatase levels, while bilirubin values remained within normal limits, making biliary pancreatitis unlikely (Table 1). Hypertriglyceridemia-induced pancreatitis was also excluded, as serum triglyceride levels were within the normal range (Table 1). These findings supported the diagnosis of acute pancreatitis secondary to severe hypercalcemia of probable malignant origin. Although the patient experienced systemic deterioration, organ dysfunction was primarily related to malignancy-associated metabolic derangement rather than pancreatitis itself; therefore, the case was consistent with moderately severe acute pancreatitis according to the Revised Atlanta classification [8]. Intensive intravenous hydration with 0.9% sodium chloride, analgesia, and bowel rest were initiated, along with further investigation for an underlying primary malignancy.

**Table 1:** Laboratory Findings at Admission AST: Aspartate Aminotransferase; ALT: Alanine Aminotransferase; GGT: Gamma-Glutamyl Transferase

Parameter	Result	Reference Range
Amylase	248 U/L	30-110 U/L
Lipase	843 U/L	13-60 U/L
Total Calcium	16.4 mg/dL	8.5-10.5 mg/dL
Ionized Calcium	2.15 mmol/L	1.12-1.32 mmol/L
Hemoglobin	9.7 g/dL	12-16 g/dL
Platelet Count	54 × 10³/µL	150-400 × 10³/µL
Creatinine	1.50 mg/dL	0.6-1.2 mg/dL
C-Reactive Protein	81.5 mg/L	<5 mg/L
Lactate Dehydrogenase	485 U/L	135-225 U/L
AST	128 U/L	10-40 U/L
ALT	30 U/L	7-56 U/L
GGT	123 U/L	9-48 U/L
Alkaline Phosphatase	366 U/L	44-147 U/L
Total Bilirubin	1.07 mg/dL	0.2-1.2 mg/dL
Direct Bilirubin	0.68 mg/dL	0-0.3 mg/dL
Indirect Bilirubin	0.39 mg/dL	0.2-0.8 mg/dL
Triglycerides	130 mg/dL	<150 mg/dL

During hospitalization, hypercalcemia persisted, with progressive hematologic deterioration. PTH level was within the normal range, ruling out primary hyperparathyroidism. PTHrP and vitamin D metabolites were not measured, as the clinical and biochemical profile strongly suggested malignancy-related hypercalcemia, and their assessment would not have influenced management decisions. Beta-2 microglobulin was elevated; however, serum free light chains were normal, and serum protein electrophoresis showed no monoclonal spikes (Table 2). Hematology consultation excluded multiple myeloma. Bone marrow aspiration was performed early, prior to other diagnostic approaches, due to hematologic abnormalities and diffuse osteolytic lesions, which initially raised a strong suspicion of hematologic malignancy.

**Table 2:** Laboratory Evaluation During Hospitalization

Parameter	Result	Reference Range
Total Calcium	14.9-15.2 mg/dL	8.5-10.5 mg/dL
Hemoglobin	8.6 g/dL	12-16 g/dL
Platelet Count	54 × 10³/µL	150-400 × 10³/µL
Parathyroid Hormone (PTH)	30.3 pg/mL	15-65 pg/mL
Beta-2 Microglobulin	6.3 mg/L	0.7-1.8 mg/L
Kappa Light Chain	1.14 g/L	0.33-1.94 g/L
Lambda Light Chain	0.90 g/L	0.57-2.63 g/L
Kappa/Lambda Ratio	1.27	0.26-1.65

Due to persistent hypercalcemia, intravenous zoledronic acid 3 mg (dose adjusted for renal function) was administered, achieving a decrease in serum calcium to normal levels; hemoglobin and platelet counts also improved; however, inflammatory markers remained elevated, including C-reactive protein (CRP) and lactate dehydrogenase (LDH) (Table 3).

**Table 3:** Laboratory Response After Zoledronic Acid

Parameter	Result	Reference Range
Total Calcium	9.4 mg/dL	8.5-10.5 mg/dL
Hemoglobin	8.6 g/dL	12-16 g/dL
Platelet Count	86 × 10³/µL	150-400 × 10³/µL
C-Reactive Protein	295 mg/L	<5 mg/L
Lactate Dehydrogenase	426 U/L	135-225 U/L

**Figure 1:**
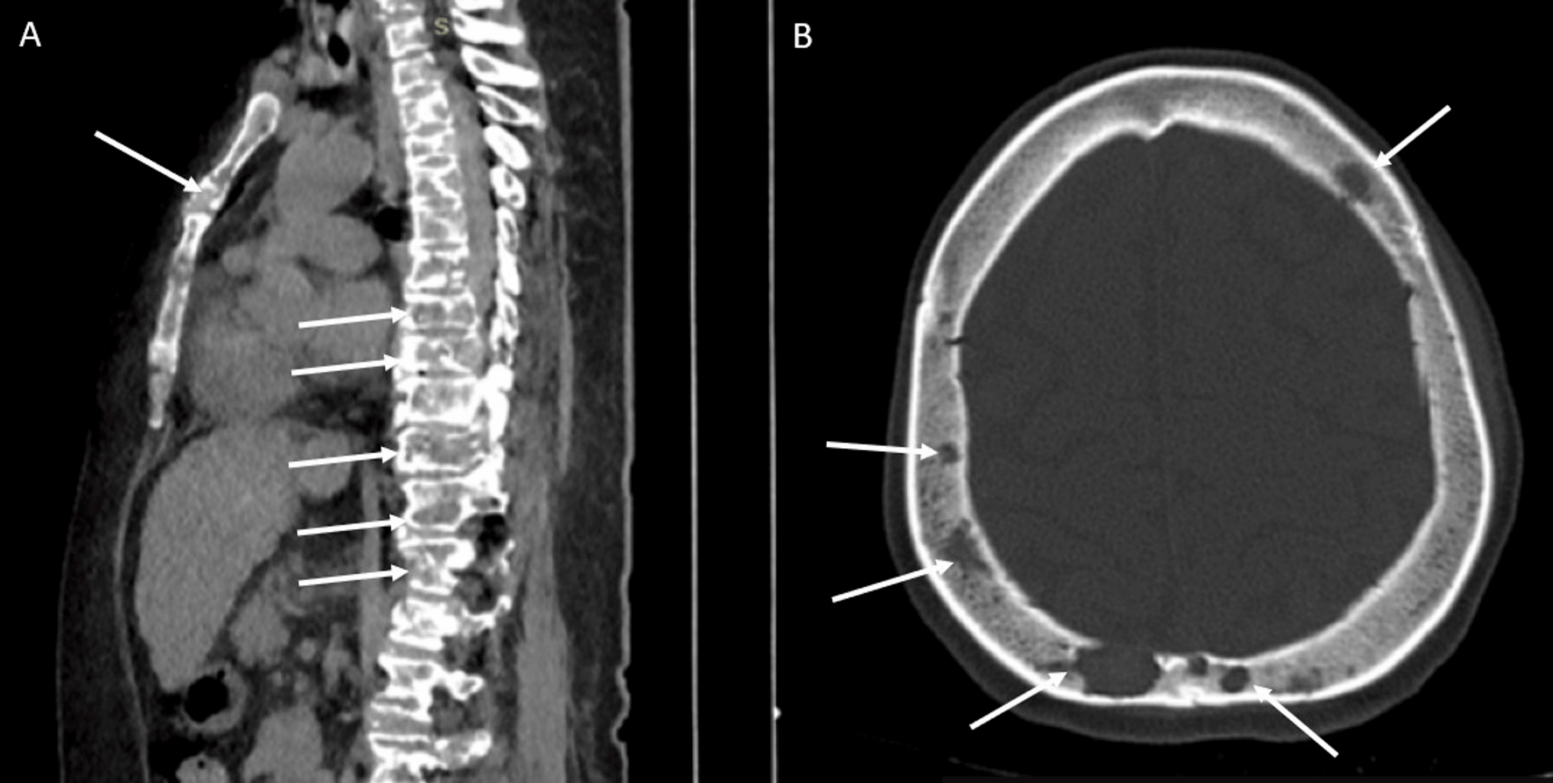
Computed Tomography (CT) Findings of Osteolytic Bone Lesions (A) Sagittal CT reconstruction showing multiple osteolytic lesions involving the vertebral bodies and sternum (white arrows). (B) Axial skull CT demonstrating multiple punched-out osteolytic lesions of the calvarium (white arrows).

**Figure 2:**
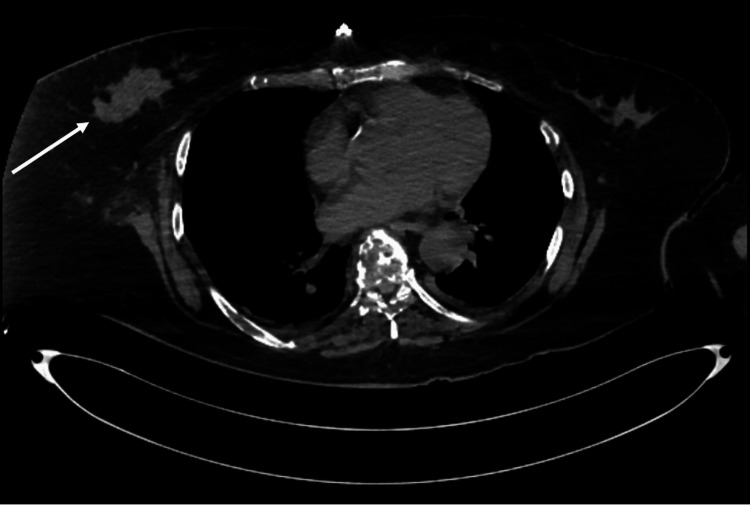
Computed Tomography (CT) Showing a Right Breast Lesion Axial chest CT demonstrating an irregular solid lesion in the right breast with irregular morphology and heterogeneous density, suspicious for malignancy (white arrow).

Computed tomography (CT) of the chest, abdomen, and pelvis revealed multiple punched-out osteolytic bone lesions (Figure 1) and retroperitoneal lymphadenopathy, initially suggestive of multiple myeloma. Contrast-enhanced CT was not performed for pancreatic evaluation, as the diagnosis of acute pancreatitis was established based on clinical presentation and biochemical criteria, in accordance with the Revised Atlanta Classification. No clinical features suggested necrotizing pancreatitis during the disease course. However, given the absence of monoclonal gammopathy, normal light chain ratio, and a “dry tap” bone marrow aspirate, alternative diagnoses were considered. The same imaging study identified a solid lesion in the right breast (Figure 2), further characterized by breast ultrasound, which showed an irregular hypoechoic mass with spiculated margins and echogenic halo, located in the upper outer quadrant (11-12 o’clock position), measuring 5 × 3 cm, with infiltrative axillary lymphadenopathy, classified as BI-RADS 5.

**Figure 3:**
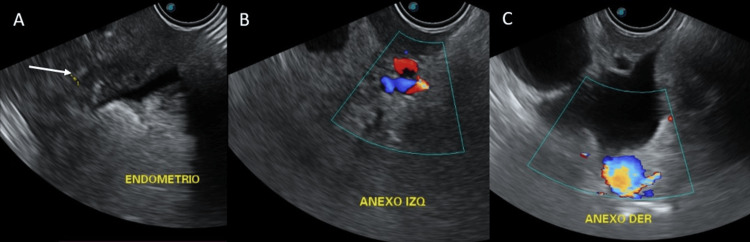
Transvaginal Ultrasound Findings (A) Thickened and echogenic endometrium (white arrow). (B) Left adnexa without suspicious findings on Doppler evaluation. (C) Right adnexa without suspicious findings on Doppler evaluation.

Tumor markers were elevated, including cancer antigen (CA) 15-3, carcinoembryonic antigen (CEA), and human epididymis protein 4 (HE4), with a ROMA (Risk of Ovarian Malignancy Algorithm) index of 94.6% (Table 4), suggesting a high risk of ovarian malignancy. However, tumor markers were supportive but lacked diagnostic specificity, and a definitive diagnosis required histopathological confirmation. Transvaginal ultrasound revealed a small uterus (6.2 × 2 × 2.6 cm) with heterogeneous texture, thickened echogenic endometrium (9.4 mm), and partially visualized adnexa without pathological findings, interpreted by oncology as an incidental postmenopausal finding (Figure 3). These findings were consistent with a malignant breast neoplasm with probable bone metastases.

**Table 4:** Tumor Markers CA: Cancer Antigen; CEA: Carcinoembryonic Antigen; HE4: Human Epididymis Protein 4

Marker	Result	Reference Range
CA 15-3	707 U/mL	<30 U/mL
CEA	404 ng/mL	<5 ng/mL
HE4	530 pmol/L	<140 pmol/L
CA 125	14.2 U/mL	<35 U/mL

A core needle biopsy of the right breast confirmed invasive carcinoma of no special type (invasive ductal carcinoma), grade 2, human epidermal growth factor receptor 2 (HER2)-positive, establishing the diagnosis of metastatic breast cancer as the cause of malignant hypercalcemia and secondary acute pancreatitis.

Despite intensive treatment with hydration, loop diuretics, and bisphosphonates, only partial biochemical control of hypercalcemia was achieved. Hematologic parameters, including hemoglobin and platelets, worsened. Additionally, although renal function normalized, hypoglycemia and severe hypokalemia developed, requiring multiple intravenous corrections (Table 5).

**Table 5:** Laboratory Findings During Late Hospital Course

Parameter	Result	Reference Range
Hemoglobin	8.6 g/dL	12-16 g/dL
Platelet Count	84 × 10³/µL	150-400 × 10³/µL
Urea	34 mg/dL	15-40 mg/dL
Creatinine	0.72 mg/dL	0.6-1.2 mg/dL
Glucose	69 mg/dL	70-100 mg/dL
Potassium	2.5 mEq/L	3.5-5.0 mEq/L

Clinically, the patient experienced progressive neurological deterioration, with a Glasgow Coma Scale score of 10/15, associated with somnolence, disorientation, and confusion, attributed to hypercalcemia and global metabolic derangement. Multiple ecchymoses were also noted. Despite therapeutic efforts, her general condition remained fragile, with decreased appetite, asthenia, and loss of functional capacity. After 25 days of hospitalization, and in view of the poor short- and medium-term prognosis, the family requested voluntary discharge. Due to rapid clinical decline and the family’s decision favoring comfort-focused care, systemic oncologic therapy was not initiated.

According to family testimony, the patient initially appeared worried and fatigued, without understanding the cause of her symptoms. Although she remained conscious and communicative during much of the hospitalization, subsequent neurological deterioration prevented her from fully comprehending the final diagnoses. Respecting the patient’s wish to remain at home, and understanding the prognosis, the family requested voluntary discharge and home-based palliative care, expressing the desire that her “final days” be spent at home. Due to neurological deterioration, the patient’s direct perspective could not be obtained.

## Discussion

Acute pancreatitis ranges from mild, self-limited episodes, with a mortality of 1%-2%, to severe necrotizing disease, with mortality rates up to 20% [1]. Gallstones are the leading cause, accounting for 35%-40% of cases, followed by alcohol consumption (17%-25%). Other causes, such as hypertriglyceridemia, drug-induced pancreatitis, endoscopic procedures, trauma, genetic mutations, and metabolic disorders - including hypercalcemia (1.5%-8% of cases) - are far less frequent and, therefore, less well documented [1,2].

Hypercalcemia is an uncommon cause of acute pancreatitis and is usually secondary to underlying disorders, most commonly primary hyperparathyroidism or malignancies - particularly multiple myeloma and parathyroid carcinoma [2,3]. Serum calcium levels above 13 mg/dL are highly suggestive of an underlying malignancy and warrant further investigation [4].

Several mechanisms contribute to malignancy-related hypercalcemia-induced pancreatitis, including excessive PTH or PTHrP secretion leading to reduced renal calcium clearance; local cytokine release from osteolytic metastases - notably in breast cancer, lung cancer, multiple myeloma, renal cell carcinoma, lymphomas, and head and neck cancers; and increased production of 1,25-dihydroxyvitamin D, particularly in lymphomas [5,9,10].

Pathophysiologically, hypercalcemia promotes intraductal activation of trypsinogen to trypsin, pancreatic tissue injury, and the formation of ductal stones. The calcium-sensing receptor (CaSR) has also been implicated, although its precise role remains incompletely understood [2,5,6].

Clinically, hypercalcemia-related pancreatitis typically presents with epigastric pain radiating to the back, along with neurological manifestations ranging from confusion to coma [1,9]. In this case, multiple myeloma was initially suspected due to anemia, thrombocytopenia, lytic bone lesions, and elevated beta-2 microglobulin; however, normal serum protein electrophoresis, normal kappa/lambda ratio, and the absence of Bence Jones proteinuria excluded a hematologic malignancy [11]. In contrast, the presence of a BI-RADS 5 breast lesion, markedly elevated tumor markers, and histopathological confirmation of invasive ductal carcinoma redirected the diagnosis toward metastatic breast cancer with osteolytic hypercalcemia.

Management of acute pancreatitis in the setting of malignancy focuses on three objectives: treating pancreatitis, correcting hypercalcemia to prevent fatal complications, and diagnosing and managing the underlying neoplasm. First-line therapy includes aggressive intravenous hydration (200-300 mL/h of 0.9% saline), calcitonin (4 IU/kg), bisphosphonates, or denosumab in refractory cases. Hemodialysis may be required in refractory hypercalcemia or severe renal impairment. Loop diuretics and corticosteroids may be indicated in selected cases [7,9]. In this patient, zoledronic acid achieved a partial biochemical response; however, persistent malignancy and hematologic deterioration led to a poor prognosis.

Hypercalcemia-associated pancreatitis is more severe than pancreatitis from other etiologies. Gieszinger et al. reported an odds ratio of 1.89 (95% CI: 1.31-2.71) for in-hospital mortality compared with other causes, with higher rates of acute kidney injury, acute respiratory distress syndrome, and cardiac arrhythmias [12]. Median survival after diagnosis of malignant hypercalcemia is one to three months and decreases as calcium levels rise. In this case, calcium levels exceeding 16 mg/dL (above the >13 mg/dL threshold commonly associated with malignancy-related hypercalcemia and poor survival) were accompanied by rapid neurological deterioration. This clinical course was consistent with the aggressive prognosis described in the literature, where higher calcium levels correlate with shorter survival and increased risk of metabolic complications [9]. The decision for voluntary discharge, made by the family after explanation of the underlying cancer, the need to start cancer-specific treatment, and the risks of omitting it - especially the fatal outcome - reflects the importance of honest and empathetic communication between the medical team and the family, especially in advanced disease scenarios where curative interventions are limited.

## Conclusions

Acute pancreatitis secondary to malignant hypercalcemia is a rare but clinically significant condition associated with poor prognosis. This case underscores the importance of considering underlying malignancy in patients presenting with pancreatitis of unclear etiology and severe hypercalcemia, particularly after primary hyperparathyroidism has been excluded. Clinicians should consider early evaluation for occult malignancy when acute pancreatitis presents with serum calcium levels ≥13 mg/dL and no alternative etiology is identified, as this threshold is strongly associated with malignancy-related hypercalcemia and may facilitate earlier diagnosis of metastatic disease. The presence of osteolytic lesions and hematologic abnormalities may initially suggest hematologic malignancies; however, solid tumors, such as metastatic breast carcinoma, should also be considered. Early recognition of hypercalcemia as a potential paraneoplastic manifestation is essential to guide diagnostic evaluation and management.

This case also highlights the importance of a multidisciplinary approach in complex presentations involving metabolic emergencies and advanced malignancy, as well as timely communication with patients and families regarding prognosis and care planning.

## References

[1] Wang CF, Tariq A, Chandra S (2025). Acute pancreatitis. StatPearls [Internet].

[2] Tiwari AK, Kumar V, Yadav DP (2022). Hypercalcemia - an enigmatic cause of acute pancreatitis. J Clin Transl Res.

[3] Imam Z, Hanna A, Jomaa D, Khasawneh M, Abonofal A, Murad MH (2021). Hypercalcemia of malignancy and acute pancreatitis. Pancreas.

[4] Anastasopoulou C, Mewawalla P (2025). Malignancy-related hypercalcemia. StatPearls [Internet].

[5] Yang L, Lin Y, Zhang XQ, Liu B, Wang JY (2021). Acute pancreatitis with hypercalcemia caused by primary hyperparathyroidism associated with paraneoplastic syndrome: a case report and review of literature. World J Clin Cases.

[6] Desmedt V, Desmedt S, D'heygere E, Vereecke G, Van Moerkercke W (2021). Hypercalcemia induced pancreatitis as a rare presentation of primary hyperparathyroidism. Acta Gastroenterol Belg.

[7] Amri F, Bensalah Y, Zazour A (2024). Acute pancreatitis related to hypercalcemia as initial manifestation of cancer: about 4 cases. Radiol Case Rep.

[8] Zerem E, Kurtcehajic A, Kunosić S, Zerem Malkočević D, Zerem O (2023). Current trends in acute pancreatitis: diagnostic and therapeutic challenges. World J Gastroenterol.

[9] Lamassab NE, Jabri M, Benzekri H (2022). Severe hypercalcemia complicated by acute pancreatitis revealing generalized bone lysis metastasis: case report and review. Radiol Case Rep.

[10] Deenadayalan V, Kumi DD, Shah M (2022). Assessing the impact of hypercalcemia of malignancy on clinical outcomes in cancer patients admitted with acute pancreatitis. J Clin Oncol.

[11] Albagoush SA, Shumway C, Azevedo AM (2023). Multiple myeloma. StatPearls [Internet].

[12] Gieszinger G, Kui B, Hegyi P (2025). Hypercalcemia causes more severe acute pancreatitis: an international multicenter cohort study. J Clin Med.

